# Editorial: Cancer, metabolism and kidney injury: from molecular mechanisms to therapy

**DOI:** 10.3389/fcell.2026.1862158

**Published:** 2026-05-07

**Authors:** Mengsi Hu

**Affiliations:** 1 Department of Nephrology, Shandong Provincial Hospital Affiliated to Shandong First Medical University, Jinan, China; 2 Department of Nephrology, Shandong Provincial Hospital, Cheeloo College of Medicine, Shandong University, Jinan, China

**Keywords:** biomarkers, cancer, kidney injury, metabolism, nephrotoxicity

## Introduction

The connection between malignant tumors and kidney injury has drawn increasing attention in recent years. The kidney’s high blood flow makes it particularly vulnerable to systemic disease, and cancer patients are exposed to renal insults from multiple sources: direct tumor effects, nephrotoxic therapies, and the metabolic and immune disturbances that accompany advanced malignancy. This Research Topic was organized to examine these overlapping problems. The eight articles collected here each address a distinct aspect of this challenge.

## Metabolic burden and cancer risk in CKD

The population-based study asks a straightforward question: how do diabetes and obesity, alone and in combination, influence cancer risk in patients with chronic kidney disease? Drawing on a nationwide Korean cohort of nearly two million adults, the authors report that both conditions independently elevate overall cancer risk, with the highest hazard ratios observed when diabetes and obesity coincide. Site-specific analyses reveal pronounced associations with gastrointestinal and female-specific malignancies. These findings argue for integrating metabolic comorbidities into cancer surveillance strategies for the CKD population.

## Prognostic models and shared biomarkers

Three articles in this Research Topic employ computational approaches to identify biomarkers and investigate disease mechanisms. Chang et al. One study focuses on the renin-angiotensin system in clear cell renal cell carcinoma, using machine learning to derive a three-gene signature (SLC6A19, SLC16A12, SMIM24) that predicts survival and correlates with immune infiltration and drug sensitivity. Functional experiments confirm that SLC6A19 suppresses ccRCC cell proliferation and invasion *in vitro*.

Chen et al. Another study examines the shared molecular basis of diabetic nephropathy and sarcopenia. By integrating multiple transcriptomic datasets, the authors identify HTT and TTC19 as mitochondrial genes common to both conditions, pointing to mitochondrial dysfunction as a plausible mechanistic link. He et al. The third study on diabetic nephropathy, which uses a similar bioinformatics framework to investigate metabolic reprogramming. The work identifies six key metabolic reprogramming-related genes; of these, CXCR2, NAMPT, and CUEDC2 were validated in clinical samples. These findings underscore the central role of metabolic dysregulation in driving renal injury in diabetes.

## Mechanistic insights and protective strategies

Zhu et al. The original research examines the renoprotective effects of GDF15 in a murine model of ischemia-reperfusion injury. Overexpression of GDF15 reduced mortality, preserved renal function, and attenuated histological evidence of injury, with corresponding decreases in pro-apoptotic and pro-inflammatory markers. The finding that GDF15 modulates circular RNA expression is intriguing, though the functional implications remain to be clarified.

Huang et al. The review addresses the context-dependent behavior of PKP1 in cancer. PKP1 resists simple classification as an oncogene or tumor suppressor. Its function depends largely on subcellular localization: membrane-associated PKP1 stabilizes desmosomes and restrains tumor progression, whereas cytoplasmic or nuclear PKP1 promotes oncogenesis through enhanced MYC translation and glycolytic reprogramming. The review also discusses the signaling pathways, including PI3K/AKT and Wnt/β-catenin, that govern this spatial switch.

## Differential diagnosis of pediatric renal masses

Kamış et al. The case report describes a pediatric patient with a renal mass initially suspected to be Wilms tumor. Histopathology could not reliably distinguish between Wilms tumor and metanephric adenoma, a rare benign entity. The diagnosis was resolved only after immunohistochemical profiling (WT1+, CD57^+^) and genetic testing confirmed the BRAF V600E mutation. This case serves as a reminder that molecular diagnostics are not ancillary; they determine management.

## Therapy-associated renal complications

Chou et al. Another case report describes a patient with advanced hepatocellular carcinoma and main portal vein invasion who developed fatal tumor lysis syndrome shortly after initiating atezolizumab plus bevacizumab. Rapid tumor necrosis triggered severe metabolic derangements and acute kidney injury that proved refractory to aggressive supportive care. The case underscores a practical point: in patients with high tumor burden, potent systemic therapy can precipitate life-threatening renal and metabolic complications, and close monitoring is essential.

## Concluding Remarks

The articles in this Research Topic illustrate the multifactorial nature of kidney injury in the oncology setting. Metabolic dysfunction, tumor-intrinsic signaling, and treatment-related toxicity all contribute. Understanding how these factors interact is essential for improving patient outcomes. We hope this Research Topic encourages further work at the intersection of cancer biology, metabolism, and nephrology. We extend our sincere thanks to all authors, reviewers, and the Frontiers editorial team for their contributions to this Research Topic.

